# Supplementary figures and images for: Diverse viral pathogens in Australian canines: limited geographic structure and the first detection of an RNA virus in dingoes
Source: Virus Evol. 2025 May 22;11(1):veaf042. doi: 10.1093/ve/veaf042 (PMC12202209; doi:10.1093/ve/veaf042)

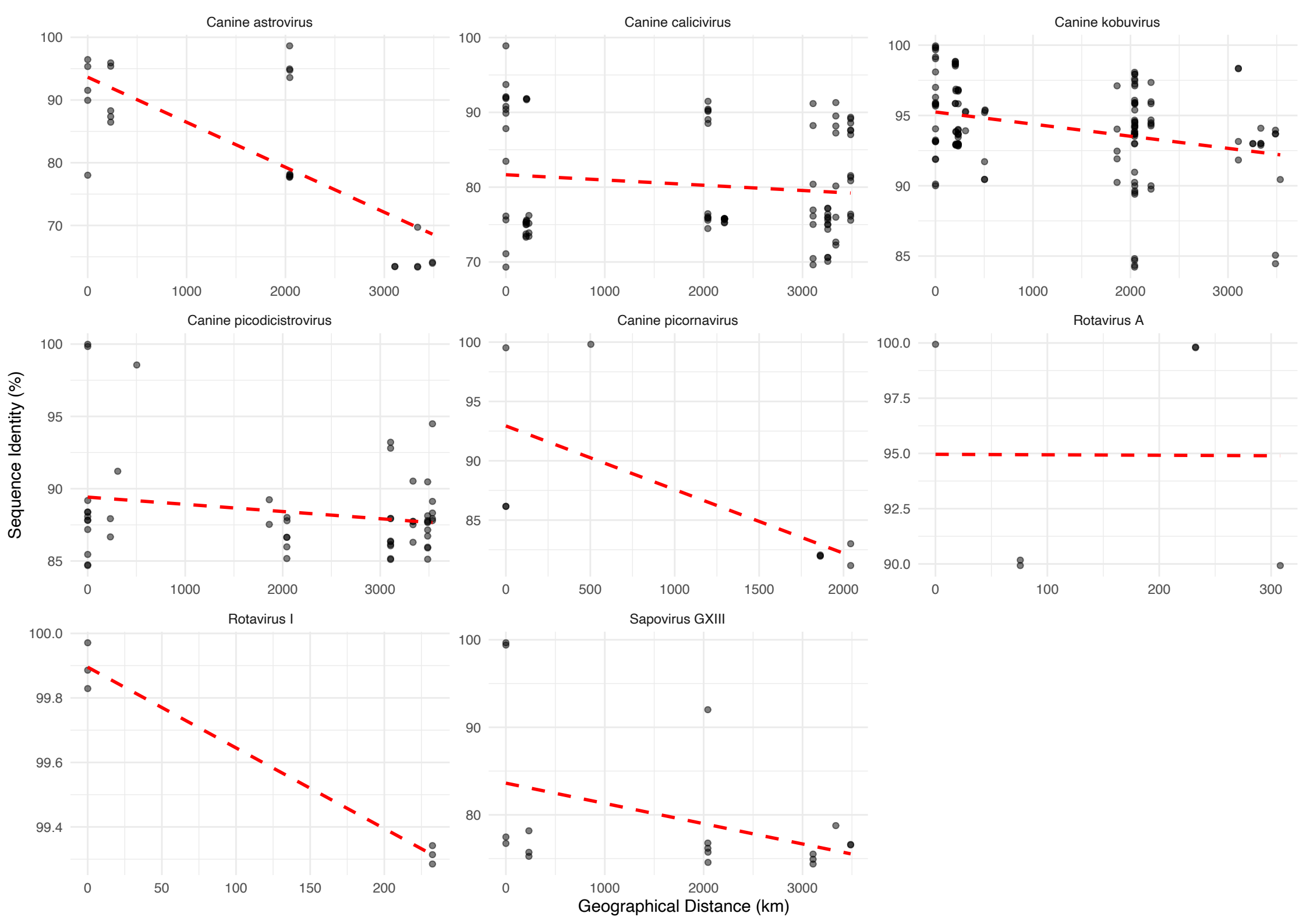

Supplement: Mifsud_Supplementary_Figure_1_veaf042 [file mifsud_supplementary_figure_1_veaf042.pdf]
