# Supplementary material for: Diverse viral pathogens in Australian canines: limited geographic structure and the first detection of an RNA virus in dingoes
Source: Virus Evol. 2025 May 22;11(1):veaf042. doi: 10.1093/ve/veaf042 (PMC12202209; doi:10.1093/ve/veaf042)
Supplement: Supplementary_Captions_veaf042 [file supplementary_captions_veaf042.docx]

**Supplementary Figures**

**Supplementary Figure 1.** Association between geographical distance and sequence identity across viral species. Each panel represents a distinct virus species as labelled, with points indicating sequence pairs plotted by geographical distance between sampling locations (km) and pairwise nucleotide sequence identity (%). The red dashed line represents the linear regression fit.

**Supplementary Tables**

**Supplementary Table 1.** Sample metadata for each library

**Supplementary Table 2.** Rotavirus A typing results

**Supplementary Table 3.** Sequence type, trimming methods, and phylogenetic models **Supplementary Table 4.** Contig statistics for each library

**Supplementary Table 5.** Summary information for the viruses identified in this study

**Supplementary Table 6.** Kruskal-Wallis test results underlying Figure 2

**Supplementary Table 7.** Dunn’s test results underlying Figure 2

**Supplementary Table 8.** Mantel test results
